# Integrating PRISM with User-centered Design (PRISM+UCD): Designing clinical decision support for safe opioid prescribing

**DOI:** 10.21203/rs.3.rs-7236339/v1

**Published:** 2025-08-13

**Authors:** Brad Morse, Katy E. Trinkley, Jason A. Hoppe, Nicole Wagner, Heather Tolle, Sarah V. Kautz, Kelly Bookman, Lisa M. Schilling, Stephen Gresham Henry, Bethany M. Kwan

**Affiliations:** University of Colorado Anschutz Medical Campus; University of Colorado Anschutz Medical Campus; University of Colorado Anschutz Medical Campus; University of Colorado Anschutz Medical Campus; University of Colorado Anschutz Medical Campus; University of Colorado Anschutz Health Sciences Building; University of Colorado Anschutz Medical Campus; University of Colorado Anschutz Medical Campus; University of California Davis; University of Colorado Anschutz Medical Campus

**Keywords:** PRISM, User-Centered Design, Opioid Prescribing, Clinical Decision Support, Implementation Science, Electronic Health Record

## Abstract

**Background:**

Balancing safe opioid prescribing with effective pain management is essential to addressing the opioid crisis. Increasing clinician adoption of evidence-based safety practices is a public health priority. This project presents a practical, adaptable approach to developing electronic health record (EHR)-embedded clinical decision support (CDS) strategies that promote uptake of opioid safety measures recommended by the Centers for Disease Control and Prevention (CDC). The goal was to design implementation strategies that enhance guideline-concordant opioid prescribing, including a suite of EHR-integrated CDS tools.

**Methods:**

Guided by the Practical, Robust Implementation and Sustainability Model (PRISM) and informed by implementation science and user-centered design (UCD), we used an iterative, multi-level engagement process. PRISM served as the theoretical framework for integrating collaborator input and user testing throughout the development cycle. Activities included discovery, design, prototyping, and usability testing. Collaborators included executive decision-makers, informatics leaders, frontline clinicians across diverse settings, representatives from the CDC and National Institute on Drug Abuse, and patients. These methods informed the selection of opioid safety measures, design themes, and workflow considerations, resulting in implementation-ready CDS prototypes.

**Results:**

The discovery phase identified a naloxone prescribing alert as a strong candidate for redesign, aligning with CDC guidelines and relevant across all four PRISM domains. The final redesign received mean ratings of 4.0 and 4.2 out of 5 on the Acceptability of Intervention Measure (AIM) from inpatient and outpatient clinicians, respectively.

**Background:**

Despite substantial public and governmental attention and investment, the opioid crisis in the United States continues to pose a serious threat to public health.^1^ In 2023, 8.6 million people across the U.S. misused opioids.^2^ In 2022, there were 81,806 opioid-related deaths in the U.S.^1^ The overall rate of unintentional overdoses was 4.3% for prescription opioids and 28.7% for any opioids.^3^ These figures underscore the urgent need for strategies that enhance safety at the point of care, specifically when clinicians prescribe opioids, while minimizing disruption to clinical workflows and ensuring applicability across diverse healthcare settings. Opioid therapy is prescribed in a wide range of clinical contexts by various types of clinicians, from primary care to specialty and emergency care. This paper outlines the process and results of co-designing clinical decision support (CDS) tools to aid clinicians in safe opioid prescribing, drawing on implementation science and user-centered design (UCD) frameworks.

## Opioid Prescribing Guidelines

The 2022 Centers for Disease Control and Prevention (CDC) Clinical Practice Guideline for Prescribing Opioids for Pain was a national effort to address the opioid crisis by improving safety in pain management.^4^ While the guidelines are not intended to replace clinical judgment, they serve as a resource and provide 12 evidence-supported care recommendations to assist clinicians to safely address pain when utilizing opioid analgesic therapy. The guidelines can be categorized into four areas: 1) decision to initiate opioids for pain; 2) selection of opioid analgesic and dosage; 3) duration of initial opioid prescription and follow up; and 4) assessment of risk and potential harms. The guidelines focus on care for patients over 18 years of age, with acute, subacute, or chronic pain and is not intended to include pain management related to sickle cell disease, cancer-related pain, palliative care, or end-of-life care.^5^

The CDC guidelines are intended to support clinical judgment and accommodate individual patient needs, rather than impose a one-size-fits-all approach. For example, Recommendation #8 advises clinicians to mitigate risk when prescribing high-risk opioids by offering naloxone—an opioid antagonist that rapidly reverses overdose. Specifically, the guideline states: “Clinicians should offer naloxone when prescribing opioids, particularly to patients at increased risk for overdose, including patients with a history of overdose, patients with a history of substance use disorder, patients taking benzodiazepines with opioids.”^4^ However, the guidelines caution against mandating naloxone prescriptions due to potential cost burdens for patients.^4^ Because the guidelines emphasize clinical discretion rather than strict requirements, tracking adherence can be challenging. Therefore, thoughtful design, implementation, and evaluation strategies are needed to promote provider uptake and assess patient benefit.

## Opioid Prescribing Guidelines Implementation Strategies

Implementation strategies refer to methods used to increase adoption, implementation, sustainability and scaling of interventions.^6,7^ CDS is one of five implementation strategies demonstrating strong evidence of effectiveness and high rates of improvement.^9–12^ CDS provides an opportunity to support clinicians within their existing workflow to inform care decisions to align with guideline recommendations and has demonstrated promise with opioid safety interventions.^8^ Importantly, CDS is often one of several discrete implementation strategies used as part of a multi-component package rather than as a standalone strategy. Other components often implemented with CDS include education, technical assistance, and/or evaluative strategies.^8^ Our CDS-based implementation strategy included redesigning workflow, developing and implementing tools for quality monitoring, and reminding clinicians at the point of decision-making. The operationalization of clinician reminders is a type of education, particularly when the reminders are related to clinical practice guidelines.^13^

Implementation strategies can be honed by incorporating various perspectives rooted in different levels within the setting where guideline concordance is sought. User-centered design is a method described by Norman and Draper in which there is a focus on the user experience instead of the technology being developed.^14^ This crucial and basic focus in the design and development of technologies that support a user has been applied in many ways.^15–17^ For example, users might be involved in usability testing to identify pain points during the development of an user interface; users might also be granted greater decision making authority in participatory design by identifying what should be developed and how.^17,18^ The application of UCD along this spectrum is commonly reported in the literature. The flexibility of UCD makes it a particularly useful and nimble method, especially when aligned with theoretical frameworks like Practical, Robust Implementation and Sustainability Model (PRISM) and used in the context of implementation science.^19,20^

The expected benefits of an implementation science framework, integrated with an UCD approach, include both technology usability as well as CDS alignment with the intervention and strategies on a multi-level scale.^21^ Consideration of the setting of where the strategies will be implemented is critical, including workflows and the multiple levels of people directly or indirectly influencing the success of the project.^22,23^ Purposeful alignment between strategies and the setting is important for building an understanding of the mechanisms of effectiveness of implementation strategies.^24,25^ The purpose of this research was to develop – and then implement and test – electronic health records (EHR)-based CDS tools for prioritized opioid prescribing guidelines. This work demonstrates a scalable, evidence-informed, process for designing CDS tools that are both usable and contextually aligned, increasing the likelihood of successful implementation and measurable impact.

## Methods

### Overview of Setting and Context

The setting was a large, university-affiliated health care system in the Rocky Mountain region with 12 hospitals, > 400 clinics, and 1.3 million outpatient and > 500,000 emergency department visits per year. The geographically diverse sites share the same EHR system (Epic: Verona, WI) with a requirement to e-prescribe all medications.^26^ The counties served cover approximately 85% of the state’s population.

The context of the project focused on the NIH HEAL Initiative^®^. Specifically, the initiative was designed to support research into adoption and implementation of the 2022 Clinical Practice Guideline for Prescribing Opioids for Pain guidelines. Helping clinicians apply these guidelines at the right time, for the right patient could be supported by implementation science, i.e., PRISM + UCD. For this project, we integrated PRISM with UCD approaches to design implementation strategies for opioid prescribing guidelines, called the PRISM + UCD approach.

Informed by UCD principles and methods and the PRISM implementation science framework, we engaged patients (patient advisory board discussion), clinicians (focus groups), and hospital leadership and administration (1:1 interviews) around design of CDS tools and other implementation strategies to support opioid prescribing guideline-based care.

### The PRISM + UCD approach

Our method comprised four UCD phases: discovery, design, prototype, and test. The integration of PRISM ensures that the resulting strategies and CDS for safely prescribing opioids are effectively integrated and sustained within specific settings and contexts.

PRISM is an adaptation to the Reach, Effectiveness, Adoption, Implementation, and Maintenance (RE-AIM) framework, a well-known implementation planning and evaluation framework.^27^ PRISM includes four domains of contextual determinants that span adoption, implementation, and sustainment of an intervention. The four domains are 1) inner setting (internal environment of the organization, i.e., culture): this domain considers patients, clinicians, and organizational leader views and experiences^28^; 2) characteristics of the individuals involved: the attributes and readiness of those who are responsible for implementing the intervention, i.e., policy makers, payers, external collaborators; 3) intervention characteristics: the resources, processes, and systems within the setting that supports continued implementation and maintenance of the intervention^29^; and 4) outer setting: this includes factors such as policies, regulations, and broader societal influences that could impact the intervention.

**Figure 1:**
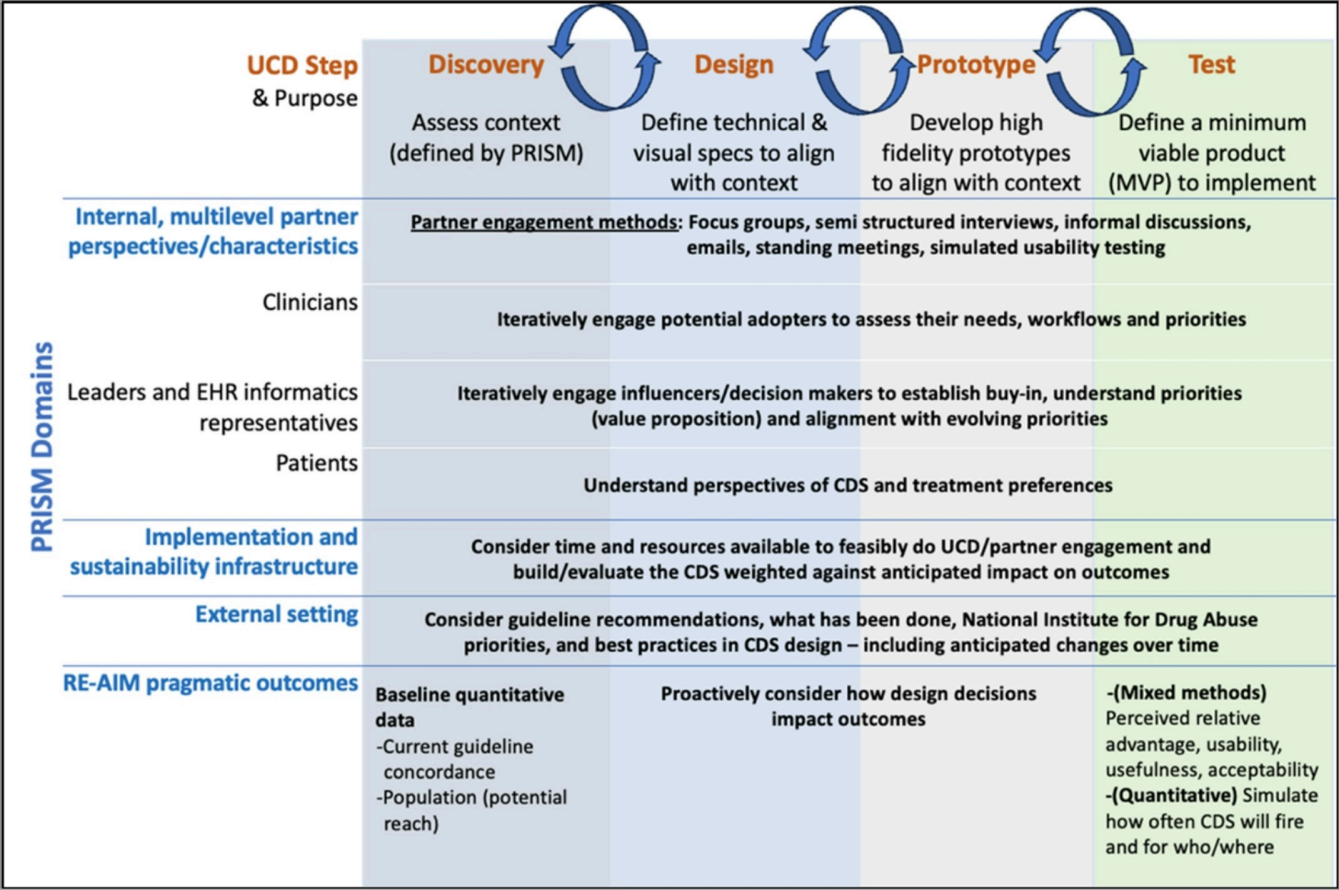
PRISM-UCD Approach to Design of Clinical Decision Support for Opioid Prescribing Guidelines

PRISM + UCD (Fig. 1) can help researchers adapt their interventions to fit local contexts and engage collaborators across phases of the project.^29^ The domains are interrelated, and data is produced for insights across the categorical domains during phase 1 of the UCD process, i.e., qualitative discovery. The support this method provides within the domains as described above. The first domain supports learning about the “perspectives on the intervention”, including both the recipient perspective as well as an organizational perspective (including leadership, managers, and frontline clinicians). The second domain provides insights on the characteristics of recipients of the intervention (both patient and organizational). The third domain is implementation and sustainability infrastructure, which includes factors such as the availability of a dedicated team that monitors fidelity to the intervention, trains clinicians on the intervention, shares best practices, and builds out an infrastructure for the sustainability of the intervention. The fourth domain is the external environment, which includes the regulatory environment of the broader health care system, the resources available to fund it, and potential alignment with health system goals.

### UCD Phase 1: Discovery

#### Patient Advisory Panel: Patient engagement

An established research advisory panel was consulted to assess patient priorities and obtain a patient perspective on opioid prescribing and safety. The panel first received an overview of the project, then provided feedback on their priorities and preferences regarding opioid use, best practices, and standardized guidelines in their medical care. We asked the following questions to assess the patient perspective: 1) What are your initial reflections on this project? 2) What aspects of this project might be of value or benefit patients who need opioids for pain management? 3) What aspects of this project might be a concern to patients? 4) What are the best ways to evaluate the outcomes of this project?

### Health System Leadership interviews

One-on-one informal interviews with health system leadership. Interviews ranged between 30 to 60 minutes and were conducted via video conferencing software^30^ and field notes were utilized. Recruitment was done through email and professional contacts.

### Clinicians focus groups

We conducted five focus groups of inpatient and outpatient clinicians (MD/DO, PA, NP) licensed to prescribe opioid analgesics in the health system. Recruitment was conducted via email contact, circulated via clinical admin/management announcement emails, and using existing EHR reports of providers recently seeing a similar CDS alert. These individuals were invited to provide input on their individual and/or clinic workflows and practices, their informational needs and preferences, as well as review the design and implementation of CDS to be integrated into the EHR system (Epic; Verona, WI). Focus group participants completed a screening questionnaire and those available during a focus group timeslot with enough other participants (6–9 participants) from the same work setting (inpatient, outpatient, or emergency department) were invited to participate. Focus groups were conducted virtually to maximize representation and assure input from across the state. Qualitative de-identified data on prescribing practices and workflow were collected.

Using a semi-structured focus group guide, participants described their current opioid prescribing workflows, prevalence of patients receiving opioid analgesics in their practice, decision processes around prescribing, and individual prescriber suggestions and needs for CDS design and development. Participants also provided feedback on ideal CDS designs in a drawing exercise. The focus groups lasted 90 minutes and were conducted via video conferencing software.^30^ All focus groups were audio recorded, transcribed verbatim with identifying information removed, and field notes taken. Thematic saturation was reached after five focus groups with clinicians.

### Discovery Synthesis

Formal thematic/contextual analysis used a matrix-style coding approach to structure analysis on key factors important to the CDS design. The focus group findings informed the design of the CDS.

### Synthesis: Affinity Grouping Method

To synthesize insights from engagement and data collection activities, the qualitative team [BM, BK, JH, KT] analyzed internal notes and focus group transcripts. Otter.ai^31^ was used to transcribe audio recordings of the focus groups. The method of analysis consisted of an affinity grouping exercise conducted in Mural^32^, an online collaboration software app. Three team members who facilitated the focus groups and the project PI conducted content analysis using a matrix-style coding approach to structure the analysis on key factors important to the CDS design. Team members reviewed a set amount of the transcripts that were assigned by the qualitative lead. Summary phrases were typed on Mural’s “sticky notes” and mapped together based on similarities in relation to five categories and the collaborator type, e.g., clinician, patient, and leadership. The five categories were as follows: 1) What guidelines are best suited for CDS? 2) What are the desired design features for the relevant CDS? 3) What are the features or elements in CDS users do not want? 4) What are the opportunities for implementation and clinical workflow? 5) What are the potential threats or barriers to the implementation of CDS? These questions facilitated the synthesis of the summaries for guidelines and desired design features and implementation strategies.

### UCD Phase 2–4: Design, Prototyping, and Testing

For UCD phase – 2, which focused on the naloxone co-prescription guideline recommendation, focus group participants referred to existing CDS and made suggestions for improvement. Then in the UCD prototyping phase – 3 these suggestions were used by the study team (KT) to create low fidelity prototypes of the CDS user-interface using Microsoft PowerPoint in which the content varied slightly for primary care versus the emergency department and inpatient settings. These prototypes were then iteratively updated with input from the study team (JH, HT, BK, BM, LS) and the health system’s clinical decision support governance. During the UCD test phase – 4, these prototypes were utilized for focused feedback circulated via email to the 67 clinicians who responded to our focus group solicitation. Feedback was collected using Qualtrics in which participants provided input on specific design features such as response options (e.g., include a ‘comment’ or ‘other’ option), duration of suppressing a CDS based on clinician response, and content displayed within the user interface.^33^ Participants were also asked to complete the 4-item Acceptability of Intervention Measure (AIM), a 4-item validated measure using a five-point Likert scale from completely disagree to completely agree.^34^ Higher scores on the scale indicate more agreement with the acceptability of the alert. Open-ended responses to the survey were used to create final prototypes built in the EHR. The outcome of these methods is a minimum viable product (MVP) which was shared with analysts to build in the EHR. The study team tested the CDS in test environments of the EHR.^34^

### Implementation Research Logic Model and Implementation Strategies

Based on the outcome of the PRISM + UCD process, we developed an Implementation Research Logic Model (IRLM) tool to guide our work. An IRLM provides collaborators and implementors with a structured opportunity to discuss and align on the core components of the logic model: determinants, implementation strategies (to address facilitators and barriers), the intervention, the mechanism of action, and the outcomes.^35^ The IRLM can be applied at multiple stages to identify implementation strategies needed both within and alongside the CDS tool to maximize program impact and sustainability.^35^ We then leveraged the Expert Recommendations for Implementing Change (ERIC) taxonomy of implementation strategies.^36^ In collaboration with our multilevel team, we discussed which strategies were feasible and appropriate within the given context.

## Results

### Affinity Grouping Participants

Four patients were engaged through a pre-existing patient and family research advisory panel.^37^ Eleven hospital leadership and administration interviews were conducted. Twenty-five clinicians (MD/DO, PA, NP) participated in five focus groups (Table 1). These three distinct data collection efforts provided the data for the Affinity Grouping methodology.

### Affinity Grouping Insights

**Figure 2:**
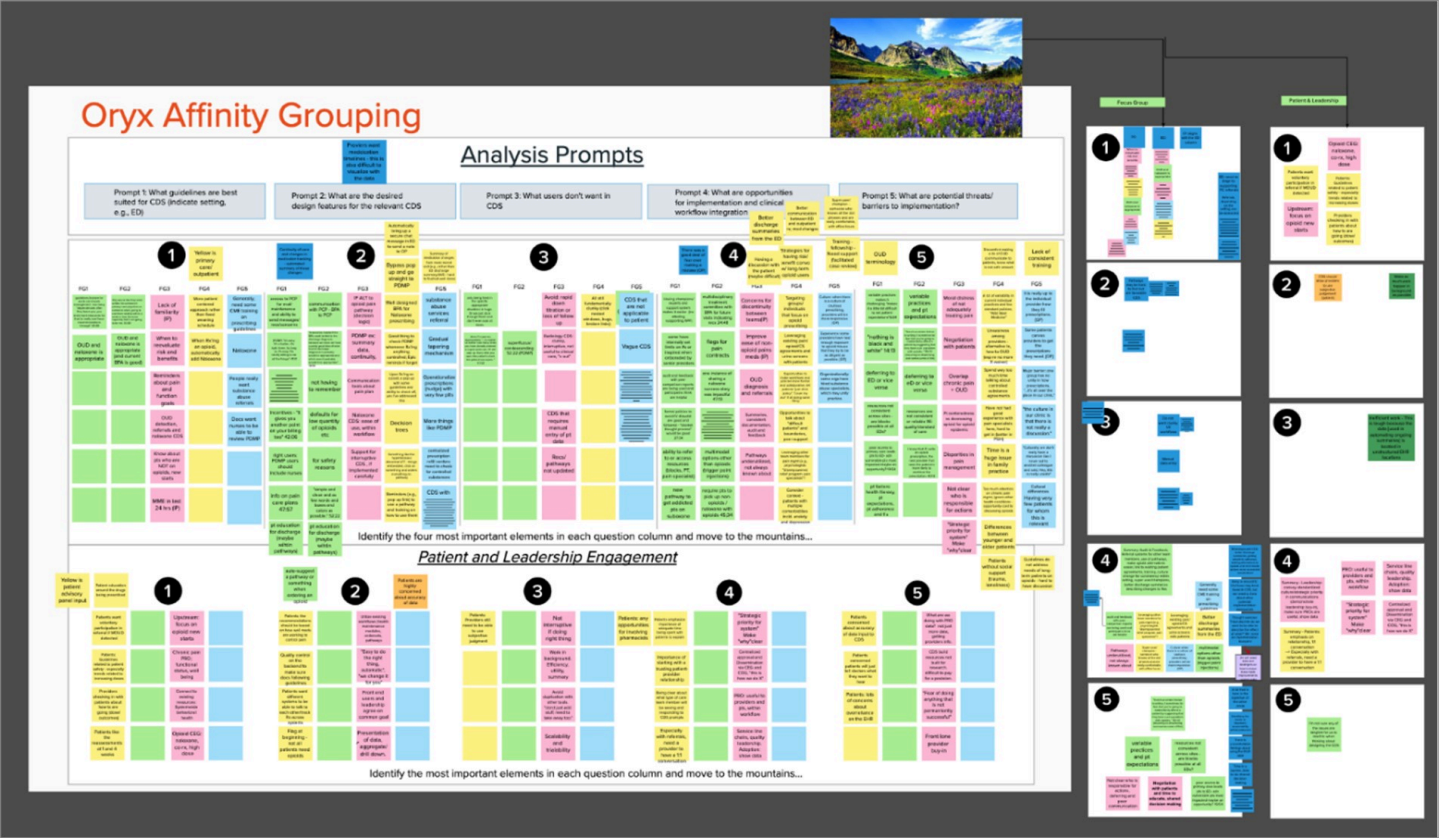
Mural board synthesis

The Mural board Affinity Grouping synthesis (Fig. 2) produced three major areas of insights. First, given the potential impact on established workflows, we found that it is easy to quickly lose users of CDS due to poor design. Therefore, streamlined workflows with accurate logic and an intuitive interface are critical. Regarding opioid prescribing guidelines and desired design features for CDS, we found that existing guidelines most suited for redesigned CDS included co-prescribing of naloxone with opioid analgesics, which is aligned with Recommendation #8. Other guidelines suited for redesign included evaluation of opioid prescribing risk and improved processes for referrals for substance abuse treatment, specifically opioid use disorder (OUD). The PRISM + UCD process revealed several key insights: 1) clinicians need timely access to information about patients’ recent prescription opioid use; 2) nurses should also have easy access to this data; and 3) CDS tools should minimize user effort by requiring as few clicks as possible and feature accurate firing logic to avoid disrupting clinical workflows.

Second, related to implementation strategies, there are several opportunities for CDS implementation and integration into clinician workflows that may support adoption by clinicians. There is an education gap for clinicians on the updated CDC guidelines. Many system clinicians reported not often interacting with the new CDC guidelines, decreasing familiarity with the new guidelines within our system. Clinicians told us that it would be helpful if individual experts were identified as domain experts for support related to opioid use disorder content, in general, and the current guidelines, specifically. Domain experts might also be local champions in the clinics. According to Leadership, clinicians want to avoid inefficient workflows. Leadership advocates for standardized culture and strategic priority in communications that demonstrate leadership buy-in, while making Patient Reported Outcomes (PRO) useful and data-driven. Patients advocate for relationships with clinicians and value 1:1 conversation, especially when a referral is involved.

Third, data from the health system leadership interviews revealed that leadership supported the project as a strategic priority for the system. They provided guidance on the approval process needed for the build and development of CDS tools within the system. Leadership expressed concern at the amount of work associated with collecting data for the sake of collecting data, stressing the need for the data to be useful to clinicians, measuring impact on workflows and outcomes, and be integrated into the workflow. Leadership also suggested that our team take a wider view of hospital activities across the system to avoid duplication of efforts by leveraging existing organizational structures and resources.

### UCD Phase 2–4 Participants

Fifteen of 67 clinicians responded to our Qualtrics survey in which participants provided input on specific design features.

### UCD Phase 2–4 Results

**Figure 3:**
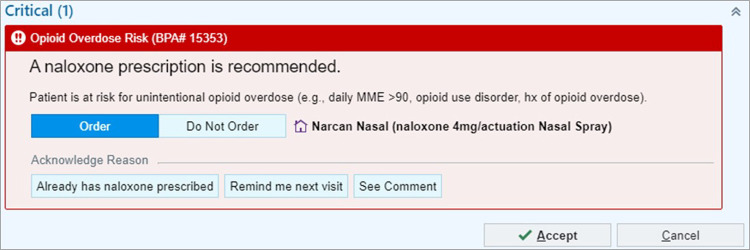
Naloxone OPA screenshot. © 2025 Epic Systems Corporation.

CDS tools were developed utilizing insights gained from all four UCD phases. During the discovery phase – 1, utilizing resources in the hospital system, and subject matter expertise from the team, an existing alert encouraging prescribing naloxone with a high-risk opioid analgesic in the emergency department was identified and adapted to meet the contextual needs across the entire hospital system. This alert is related to Recommendation #8, as discussed in the Background. The MVP low fidelity prototype selected to be built for testing in the EHR test environment is available in Fig. 3. A total of eight inpatient and emergency department clinicians rated the naloxone alert with an average score of 4 out of 5 on the AIM. Seven outpatient clinicians rated the alert as a 4.2 on the AIM, showing high acceptability of the alert design.

### Implementation Research Logic Model

**Figure 4:**
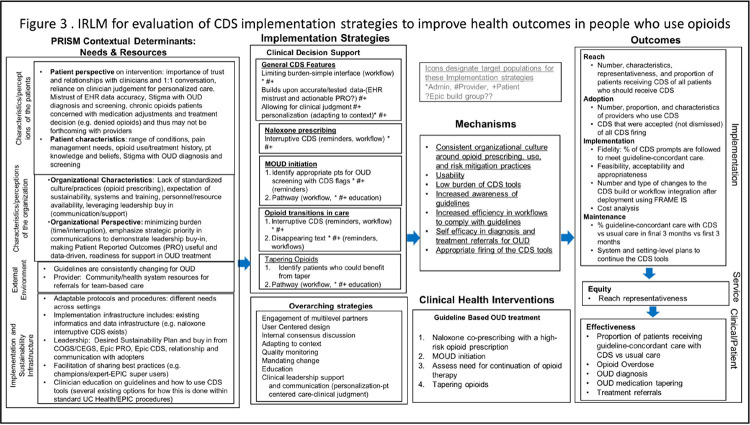
Implementation research logic model for opioid prescribing guideline implementation

Based on outputs from the Mural board synthesis, we (BK, JH, HT, KT, BM) developed an IRLM (Fig. 3). The logic model depicts PRISM-aligned contextual factors, corresponding implementation strategies – including both CDS tool features and additional strategies described in the ERIC framework – expected implementation strategy mechanisms, and RE-AIM outcomes for implementation of the opioid prescribing guidelines in a range of clinical settings. As shown in Fig. 4, we identified two main categories of implementation strategies: CDS tools and additional strategies. The additional strategies included those already being employed during the discovery and design stages of the project as well as strategies that were recommended for use during the forthcoming “test” stage of the project. The use of the PRISM + UCD approach itself represents an implementation strategy reflecting ERIC’s “engagement of multilevel partners,” “internal consensus discussions,” and “adapting to context” domains. There were *a priori* plans to include quality monitoring and mandating change strategies. Additional recommended implementation strategies were selected based on two considerations: 1) conceptual alignment with the PRISM contextual factors identified during discovery and design phases, and 2) feasibility assessments given project resources and study team experience working with the health system. For example, recommendations for champion support strategies reflected clinician preference for having a “go-to” person for sharing best practices for use of the CDS tools in their clinical context. Recommended strategies also included clinician education (on both opioid prescribing guidelines and use of the CDS tools) and clinical leadership support and communication – both explicitly requested by participants in the clinician focus groups.

## Discussion

This work highlights a novel integrated approach to designing implementation strategies by combining PRISM + UCD. This integration enhances the relevance and usability of the strategies and fills a gap in the literature by offering a practical model for designing implementation strategies that are both theory-informed and user-responsive. There are relatively few well-described methods for designing implementation strategies. Implementation mapping, implementation research logic models, and group model building are among the established methods.^38^ It is common for implementation strategy design efforts to start with identification of adoption and implementation determinants, such as those described by implementation science frameworks like the PRISM. Identification of determinants is then followed by selection and/or tailoring of discrete implementation strategy components (such as described by the ERIC project) that are thought to address those determinants.^38^ For implementation strategies and interventions that are meant to interface with technology, such as CDS tools delivered via the EHR, UCD techniques can be a useful complement to the framework-driven approach.

Using PRISM as a guide in the iterative UCD process presented an opportunity to maximize the value of CDS tools. Using a process that includes multiple levels of input from different organizational perspectives to inform the CDS design and development facilitates maximize system-level support, clinician use, positive patient-level health impact, and minimize unintended consequences (e.g. tapering guidelines association with increased heroin use).^39^

A comparison of our work that utilizes PRISM + UCD to inform the IRLM reveals a more representative approach to the standard manner by which CDS is traditionally developed. Traditional approaches tend to be top down, not taking into consideration the users of the CDS and how they respond to prompts, nudges, and alerts as clinicians, but also users of an electronic interface that is tedious with clicks.^40,41^ Additionally, traditional approaches do not incorporate iterative approaches into the design process and may not take into consideration the context in which the CDS is being implemented.^42^ Other previous approaches have incorporated UCD that included user involvement and iterative design, which provided rich feedback and an attempt to incorporate this feedback into subsequent iterations.^43–46^ However, without the presence of the PRISM framework, the assurance of all relative perspectives cannot be guaranteed. Additionally, at a high level, the projects all took a multidisciplinary approach by generally involving diverse collaborators, although the systematic organization of the collaborates was not as refined as what PRISM supports.

Our approach combines PRISM + UCD and capitalizes on contextual factor integration at multiple organizational levels. A core strength is that this approach remains nimble and adaptive, which makes space for the UCD element by focusing on and magnifying the expertise and skillsets of vested collaborators who can speak to CDS, and implementation needs. An example of the pragmatic approach we took is the relatively short period of time we were able to successfully design and build the naloxone alert CDS tool in seven months, including three months of PRISM + UCD work, and four months of analyst build time in the local EHR. Simultaneously, we also designed implementation strategies for the CDS in the local health system, which is included in the IRLM.

The next steps of this project will be the implementation of the CDS in the local healthcare system. Outcomes of interest are captured in the IRLM, and include Reach, Adoption, Implementation, and Maintenance. All these outcomes will be a good measure of the naloxone CDS strategy built with a PRISM + UCD methodological approach that attempts to take into consideration the full reality of the setting and context in which the strategy is embedded.

### Limitations

One limitation is that there is bias introduced into the study by working with voluntary, self-selected focus groups. Another limitation is that there could be a disconnect between what clinicians are reported valuing in theory and in practice.^47^

## Conclusions

We developed and applied an integrated, rapid-cycle approach to designing CDS tools for safe opioid prescribing across inpatient, emergency, and outpatient settings in a large regional health system. Guided by the PRISM framework and UCD, we identified CDC guideline-aligned safety measures, redesigned a naloxone alert, and produced an implementation-ready CDS tool that received strong usability ratings from clinicians. This work illustrates how combining implementation science with UCD can accelerate the development of context-sensitive, evidence-based CDS strategies. The resulting alert was not only technically feasible but also endorsed by clinical leadership and supported by targeted education and communication strategies to encourage adoption.

This integrated methodology offers a scalable model for developing CDS tools that are both clinically relevant and operationally sustainable. Future work will focus on evaluating the real-world impact of the naloxone alert on prescribing behavior and patient outcomes, as well as adapting this approach to other guideline-based interventions. Continued collaborator engagement and iterative refinement will be essential to ensuring long-term success and broader dissemination.

## Figures and Tables

**Table 1:** Demographics of focus group participants and qualifying clinicians responding to recruitment

	Participated in FG N (%)	Completed Screening N(%)
Gender		
Man	13(52.0)	23(34.3)
Woman	12(48.0	44(65.7)
Self-identify	0(0.0)	0(0.0)
Age		
20–29	0(0.0)	4(6.0)
30–39	13(52.0)	27(40.3)
40–49	7(28.0)	22(32.8)
50–59	4(16.0)	13(19.4)
60 and over	1(4.0)	1(15)
Clinician Type		
MD/DO	23(92.0)	49(73.1)
NP/PA	2(8.0)	18(26.9)
Work Setting		
Emergency Department	10(40.0)	20(29.9)
Inpatient	7(28.0)	18(26.9)
Other	3(12.0)	5(7.5)
Outpatient	5(20.0)	24(35.8)

## Data Availability

Data sharing is not applicable to this article as no datasets were generated or analyzed during the current study.

## Abbreviations

EHR: Electronic Health Record
CDS: Clinical Decision Support
CDC: Center for Disease Control and Prevention
PRISM: Practical Robust Implementation and Sustainability Model
UCD: User Centered Design
AIM: Acceptability of Intervention Measure
RE-AIM: Reach, Effectiveness, Adoption, Implementation, and Maintena
MVP: Minimum Viable Product
IRLM: Implementation Research Logic Model
ERIC: Expert Recommendations for Implementing Change
OUD: Opioid Use Disorder PRO – Patient Reported Outcomes
